# Analysis of Land Use/Land Cover Change and Its Implication On Natural Resources of the Dedo Watershed, Southwest Ethiopia

**DOI:** 10.1155/2022/6471291

**Published:** 2022-09-20

**Authors:** Mesfin Anteneh

**Affiliations:** Bahir Dar University, Department of Geography and Environmental Studies, Bahir Dar, Ethiopia

## Abstract

This study analyzed the land use/land cover (LULC) change and their causes and implications on the natural resources of the Dedo Watershed, Ethiopia. The study used 1984, 2000, and 2017 satellite images to detect the trends of land use/land cover change. Moreover, key informant interviews and focus group discussions were used to identify the driving forces linked to the changes and their impact on the natural resources of the watershed. The study identified five LULC types as follows: cultivation, settlement, dense forest, light vegetation, and grassland. Over the last 33 years, cultivation and settlement land expanded by 15.7% and 0.34%, whereas dense forest, light vegetation, and grazing land declined by 9.2%, 4.97%, and 1.85%, respectively. The establishment of the resettlement program and population pressure and associated demands were the major driving forces behind the land use/cover change. Whereas increased soil erosion, biodiversity loss, and decline in water resources are identified as the major impacts of land use land cover changes in the study watershed for the last 33 years. The study concludes that if these trends of cultivation and settlement land expansion allowed continuing, there will be no dense forest vegetation will remain. So, the finding of this study is significant.

## 1. Introduction

Land use/land cover change (LULCC) is the modification of the Earth's terrestrial surface caused by human activities [1–3]. The LULCC process has a negative impact on biodiversity, climate, soil, and air, as well as the ecosystem in general, and it has become the most serious environmental concern for humans in recent years [4–6]. LULCC can be used to assess ecosystem changes and their environmental implications at various temporal and spatial scales, making it useful for understanding environmental changes [7].

Land use/land cover changes (LULCCs) are triggered by the interplay of socioeconomic and natural environmental factors. Inappropriate farming practices, overgrazing, rapid growth in the human population [8–12], and weak institutional setup [9] are among the key anthropogenic driving variables of LULCCs. Rapid changes in the number of human populations initiate to the encroachment of farming and grazing on the fragile surface topography. Advances in technology and weak institutional response on the other hand promote uncontrolled lumber cutting and overuse of communal mountain resources that further encourage increased land degradation and LULCCs. Climate variability on the other way influences the succession of plant and animal species over the fragile mountain ecosystems.

Over the last decades, a number of studies conveyed the occurrences of LULC changes, their causes, and the resulting natural resource degradation in different parts of Ethiopia [13–24]. These studies indicated a loss of natural vegetation cover, an expansion of cultivated land, and an increase in land degradation. Thus, there can be impacts on the livelihoods of local communities. Nevertheless, there is a significant variation in terms of the level of analysis, purpose, and outcome of these studies. In addition, there are also differences in terms of the geographical location and characteristics of these case studies. The spatial heterogeneity thus leads to variability in the presentation of the causes and processes of LULC dynamics. Therefore, area-specific information on LULC dynamics is essential for land-use planning aimed at appropriate resource management and maximizing the productivity of agricultural and non-agricultural land, on both local and regional levels [25]. The overall purpose of this research paper is to investigate the available knowledge and scientific information about the magnitude and rate of LULC change and its impact on the degradation of natural resources in the Dedo Watershed.

## 2. Materials and Methods

### 2.1. Description of the Study Area

**Figure 1:**
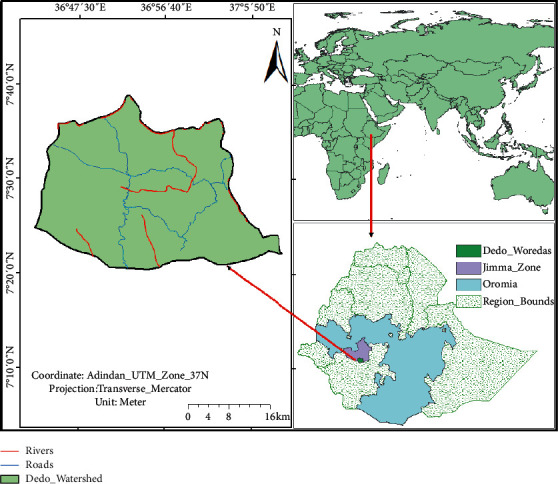
Map of the study area.

The study was conducted in the Dedo *watershed* which is, located in the Jimma Administrative Zone of Oromia National Regional State at about 340 km southwest of Addis Ababa, the capital of Ethiopia. Geographically, it is located between 7°5′ to 7°45′ N and 36°39′ to 37°15′ E (Figure 1). The watershed comprises a total of 30 kebeles (the smallest administrative unit of Ethiopia) and an area of 1094 km^2^ [26]. According to the 2007 census, the total population of the watershed was 156,987 [26]. The study area is generally characterized by semihumid highlands. The mean annual temperature of the region varies from 17°C to 24°C. The area receives monomodal rainfall of approximately 1000 to 1500 mm per year, the majority of which falls between June and August.

Subsistence mixed (crop and animal) agriculture is the major means of livelihood in the area with an average farm size of about one hectare (ha). The common growing crops are maize (*Zea mays* L.), *teff* (*Ergarostis teff Zucc*), rice (*Oryza galberrima*), and beans (*Phaseolus vulgaris* L.). Other less important crops include chickpea (*Cicer arietinum*), potato (*Solanum tuberosum*), onion (*Allium cepa*), cabbage (*Brassia aleracea*), and *Chili pepper* (*Capsicum* spp.). Various types of crops such as garlic (*Alliium sativum*), Ethiopian mustard (*Brassica carrinata*), oats (*Avena sativa*), and carrot (*Dancus carota sativus*) are also cultivated as cash crops. Domestic animal such as cattle, goats, sheep, donkeys, chickens, and bees are kept on a traditional basis.

### 2.2. Data Collection and Analysis

This study uses three different sets of Landsat satellite images for the Dedo watershed over three decades (1984 to 2017). The satellite images were acquired from the USGS NASA website (https://www.earthexplorer.usgs.gov) in GeoTIFF file format projected in UTM Zone 37° North and with WGS 84 datum and coordinate system. The three Landsat satellite images with 30 m spatial resolution were obtained for the years 1984, 2000, and 2017. In the study area, in the year 2000, there was a policy change in land use and redistribution of lands for different land purposes. Thus, this year was used as a benchmark to see the dynamics of land use land cover 15 years back (1984) and after (2017) was taken. Besides, the availability and quality of the image have been also considered to select the specified years. Dry season and cloud-free images were used since analysis are easier the images were acquired in the same season to avoid the effect of seasonal variation and the month of January was selected to get cloud-free images and real land cover features [18]. Though the images are geometrically corrected, radiometric correction and image preprocessing such as sub-setting and layer stacking were performed before the commencement of the actual classification. All the acquired satellite images were enhanced using histogram equalization and haze redaction to improve image quality. *In situ* data (ground truth points) were collected from the field for image classification and accuracy assessment. In order to identify the major driving forces of LULC changes and other nonvisual information that could not be extracted from the satellite images, focus group discussions (*n* = 3), with 4 to 6 participants from the local elderly, local community leaders, and agricultural extension workers were organized. In addition, key informants (*n* = 24) from the local people were interviewed to receive additional insights on issues related to LULC changes.

### 2.3. Data Analysis

A pixel-based supervised image classification with a maximum likelihood classification algorithm was used to classify the land-use/cover types of each reference year [27]. Ground truth points were collected from the field for the year 2017 and for the historical images (1984 and 2000) elders' interviews, pre-existing maps, aerial photos, and Google Earth were used to collect reference points for classification and accuracy assessments. A minimum of about 30 random ground points per class were used for classification and accuracy assessment [28].A similar approach has been used to study LULC change in the Libo-kemkem district of the Ethiopian highlands [29]. The accuracy assessment points were independent of those used as training samples. Accuracy assessment was carried out by creating an error matrix. The matrix compares information obtained by reference sites to that provided by classified images for a number of sample areas. Accordingly, overall accuracy, producer's and user's accuracies, and Kappa statistic were calculated from the error matrix for each reference year using the equation [30]as follows:

(1) $$\begin{array}{c} K=\frac{N\sum_{\mathrm{i=1}}^{r}X_{\mathrm{ii}}-\sum_{\mathrm{i=1}}^{r}\left(x_{i}+\left(\mathrm{x+i}\right)\right.\;}{N^{2}-\sum_{\mathrm{i=1}}^{r}\left(x_{i}+\left(\mathrm{x+i}\right)\right.}, \end{array}$$

where *r* is the number of rows in the matrix, *X*_*ii*_ is the number of observations in row *i* and column *i*  (the diagonal elements), *x* + *i* and *x*_*i*_+ are the marginal totals of row *r* and column *i*, respectively, and *N* is the number of observations.

Then, the LULC changes between two periods (i.e., 1984 to 2000 and 2000 to 2017) were quantified. Change analysis was conducted using the postclassification image comparison technique, which was used in order to minimize possible effects of atmospheric variations and sensor differences with spatial resolution [2]. Images of different reference years were first independently classified, and afterwards, change detection processes were performed. The percentage of land use/land cover change detection was made using the following formula [18]:

(2) $$\begin{array}{c} \text{Percentage LULC Change}=\frac{\text{Area final year}-\text{Area initial year}}{\text{Area initial year}}\times 100,\\ \mathrm{LUI}=\frac{U_{b}-U_{a}}{U_{a}}*\frac{1}{T}*\mathrm{100}\%, \end{array}$$

where LUI = the annual rate of change in area for the land use classes. *U*_*a*_ = area of land use class at time *a*, *U*_*b*_ = area of land use class at time *b*, and *T* = length of time in the year between *a* and *b*. If **LUI** < **0**, the land cover type is in a state of depletion. The larger the absolute value of **LUI**, the more intensively land has been depleted. **LUI** ≥ **0** means just the opposite (the land cover type in a state of expansion) [31]; Zhang et al., 2015).

Hence, positive values suggest an increase whereas negative values imply a decrease in the extent of LULC. In addition, a change detection matrix of “from-to” was derived to show LULC class conversion transitions during the 33-year period by overlaying the 1984 and 2017 classified maps. Image classification accuracy assessments and change analysis were undertaken in ArcGIS10.3 software. The datasets collected about the causes of LULC changes from FGD and key informants were analyzed qualitatively and triangulated with secondary data and the result of LULC. Based on this information, five land use classes were identified (Table 1).

## 3. Result and Discussion

### 3.1. Land Use/Cover Classification

**Figure 2:**
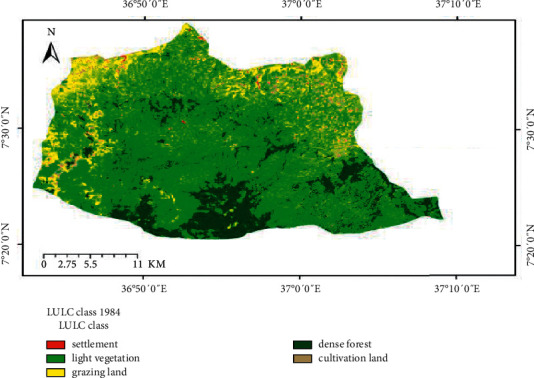
Land use land cover map of 1984.

**Figure 3:**
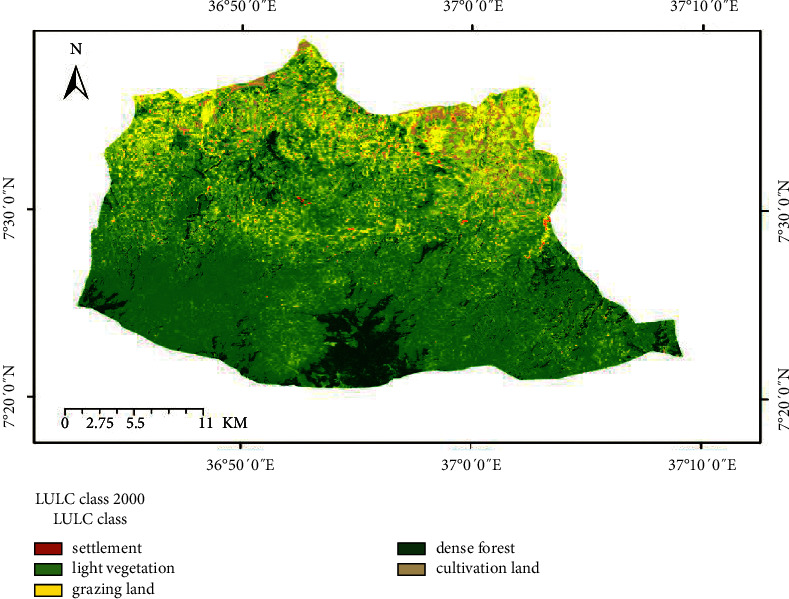
Land use land cover map of 2000.

**Figure 4:**
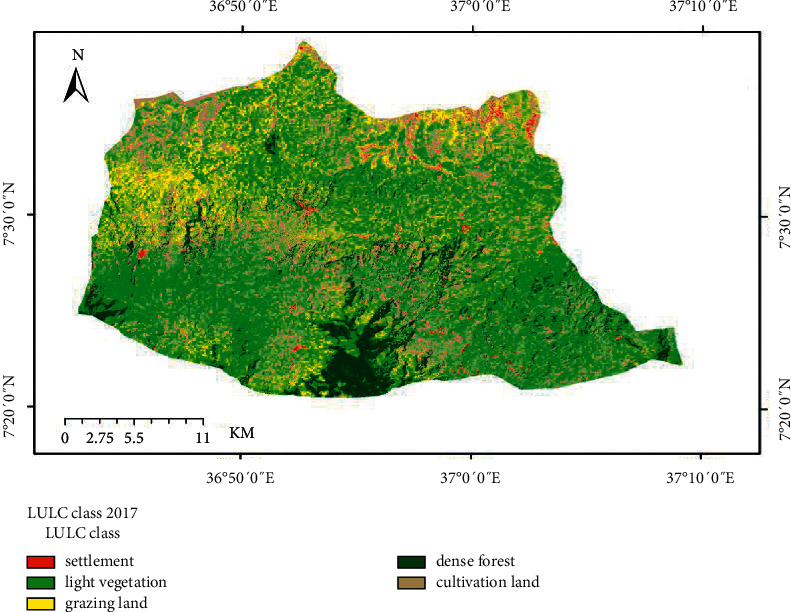
Land use land cover map of 2017.

The land use/cover trend analysis made for the two consecutive periods 1984 to 2000 and 2000 to 2017 has indicated that the watershed was exposed to considerable land use changes (Figures 2–4). The conversion of dense forest, grazing, and light vegetation land to cultivation land was the major change observed in the study period.

#### 3.1.1. Cultivation Land

During the entire period, the area under cultivated land increased persistently from 21.4% (21913.56 ha) in 1984 to 28.2% (28925.48 ha) in 2000 and 37.1% (37991.28 ha) in 2017 (Table 2). The trends showed a consistent expansion of cultivated land over the decades being considered (1984 to 2017). Accordingly, there was an increase of 6.84% (7011.92 ha) between 1984 and 2000 and 8.84% (9065.8 ha) between 2000 and 2017 (Table 3). As was outlined by the participants of the focus group discussion, the relatively smaller decline observed in this land use type during the first study period was because of the fact that farmers were forced to abandon and leave their cultivated land for settlement purposes due to the resettlement program which was undertaken since 1986. As the detection analysis results revealed, the observed consistent expansion was attributed to the conversion of the grazing land, light vegetation land, and dense forest cover into cultivated land at different stages. For instance, between 1984 and 2000 the cultivated LULC category exhibited a net gain of 14.7% of dense forestland, 11.6%% of grazing land, and 7.47% of light vegetation cover, while cultivated land maintained about 94.22% of the original size to remain under the same land use category (Table 3). This shows a consistent increase in the expansion of cultivated land. Similarly, in the period between 2000 and 2017, the land under cultivation achieved a considerable net gain of 17.01% from grazing land, 23.64% of dense forest land, and 10.52% of light vegetation land (Table 3). On the contrary, the conversion of cultivated land to the other LULC classes was relatively insignificant.

#### 3.1.2. Grazing Land

This land use type in the watershed was found in two forms; a smaller piece of private land around homesteads and farm sides reserved for grazing, and extensive communal grazing fields with free grazing away from home mainly in valley bottoms and plains. The trend analysis in this land use showed a continuous decline in the entire period considered. The first period, 1984 to 2000, was characterized by a slight decline in grazing land use from 17.6% to 16.68% of the watershed area mainly due to the coinciding radical expansion of cultivated land use, from 21.4% in 1984 to 28.22% in 2000. Despite the rapid decline in dense forest cover from 22.48% to 17.95%, and conversion into agricultural fields, grazing land areas have shown a considerable decline as opposed to the increment in cultivated and settlement land use. Similarly, in the period between 2000 and 2017, the land under grazing land has shown a declining trend with a value of 17097.78 ha to 1615 ha, respectively This can be attributed to the rapidly growing demand for cultivated and settlement land for other land uses in that specific period.

#### 3.1.3. Settlement Land

The settlement category result showed there was a change in coverage or settlement expanding from 1984 to 2017. Statistically, the area used for settlement in 1984 was 0.37% (380.21 ha) and has increased by 180.65 ha and covered 0.55% (560.85 ha) in 2000. In 2017 the area covered by a settlement reached 0.71% (729.18 ha) increasing 168.25 ha from the former year. The primary reason for such an increment of settlement area is again the need for fast population growth to acquire land for shelter as stated by the local community and focus group discussants.

#### 3.1.4. Light Vegetation Land

A continuous decline of light vegetation land cover was observed over the study period. Out of the total area of the watershed in 1984, light vegetation land constituted about 38.15%. In 2000 and 2017, it accounted for 36.6% and 33.2%, of the total area of the study watershed, respectively (Table 2). This reveals the loss of light vegetation land cover between 2000 and 2017 is high as compared to the period between 1984 and 2000. This decline in the loss of light vegetation land cover was due to the reforestation efforts initiated by the regional government since 1980 in the study site. However, the net loss of light vegetation land cover over the entire analysis period was 5085.88 ha (5%), and the largest conversions were made into cultivated and grazing land cover, respectively (Table 3). On the other hand, the gains in this category from other land use and land cover types were small as compared to the amount lost; thus, the total area under light vegetation land showed a big change over the entire period considered. The increased demand for cropland and cutting of shrubs for fuel wood was the apparent causes for the observed spatiotemporal change of light vegetation land cover.

#### 3.1.5. Dense Forest Land

The land use land cover change analysis revealed that the area under dense forest cover declined from 22.48% in 1984 to 17.95% in 2000 and finally remained at nearly 13.27% after 17 years in 2017. About 14.17% (3265.28 ha) and 7.17% (1651.76 ha) of this land cover type was converted into cultivation and light vegetation land, respectively, between 1984 and 2000, whereas in the second period, nearly 23.64% of the dense forest cover was converted into cultivation land. According to FGD participants, the major reasons for the decline in forest cover were the expansion of agricultural (crop and livestock) production activities, and the cutting of trees for timber, fuel wood, and charcoal production, in line with this Assefa [32] indicated that the decline of forest cover is mainly caused by expansion of agricultural land and cutting of trees for house construction and charcoal production.

### 3.2. Causes of Land Use Land Cover Change

Based on the results of key informants and focused group discussions (FGDs), LULC changes in the study watershed were caused by various factors. However, the establishment of the resettlement program and the ever increasing of population pressure were among the major ones. These causes are also reported in regional and national documents (e.g., Bureau of Agriculture [33]).

#### 3.2.1. Resettlement Program

The resettlement program was mentioned by the FGD participants and key informants as one of the important factors behind the changes in the study area. They described the resettlement program that took place in the 1980s in the study watershed led to the clearing of forests to acquire new agricultural and settlement land. They also mentioned that as a result of the resettlement program there was huge loss of forests within which indigenous trees were removed due to selective logging for house construction, fuel wood, and expansion of crop production. *Juniperous procera*, *Millettia ferugunea,* and *Ximenia ameriicana*, and species of *Acacia* were among the woody species that were seriously affected. The thousands of homes in the Dedo watershed and its surroundings were also good indicators of the extent of the forest destruction. For example, there were about 1870 housing units in 1986 [35]. This figure grew to 4,058 homes in 2009 [26]. This increase in the number of human settlements was at the expense of the natural vegetation, which was used for construction materials and residential space.

#### 3.2.2. Population Pressure

Change in population size, distribution, and associated demographic characteristics are often considered the most important factors affecting land use distribution and change [36, 37]. Accordingly, population of the study area increased from 47,005 in 1994 [35] to 65,129 in 2007 [26]. Based on the data from the district office, the population further increased to 96,275 in 2016, making the population of the study area doubled over the past 20 years. Such rapid population growth in the area has already exerted pressure on the existing land resources by increasing the demand for food, wood for fuel and construction material purposes, and other necessities, as is also reported in earlier studies (e.g., [23, 38, 38].

### 3.3. Implications of the Observed LULC Change on Land Resources

#### 3.3.1. Implications of Soil Erosion

Land use/cover change is one of the most important factors that govern the surface runoff, rate of soil erosion, and sediment yield from the catchments [39, 40]. The ever-increasing deforestation that happened in the watershed for decades due to different human activities especially for settlement and crop production together with the rugged landscape has exposed the study watershed to soil erosion. According to the information obtained from the key informants, many uplands and escarpments of the watershed are exposed to different human-induced practices such as the clearing of natural vegetation for different uses, cultivation of steep slopes, inappropriate farming systems, and absence of soil conservation and soil fertility management methods were seriously affected by soil erosion. As a result, they lose much of their soil through water runoff. Therefore, the loss of natural vegetation covers, such as shrub lands and low cover of forests in the watershed and its surroundings, and their successive conversions to cultivated and settlement areas in the absence of effective soil and water conservation strategies indicated the prevalence of soil erosion. Furthermore, as confirmed by focus group discussions, intercropping is not a common practice in the area, and this also exposed the cultivated land to more erosion. This is an indicator of the need for prioritizing and developing of different conservation strategies and plans at the watershed levels, such as the Dedo watershed.

#### 3.3.2. Implication on Hydrological Regimes

The change in LULC significantly alters the hydrological fluxes such as runoff response, availability of water resources, and the environment on a local and global scale [41–43]. This change in the LULC pattern, such as deforestation and subsequent cultivation, could reduce the infiltration rate and percolation of rainwater to recharge streams, springs, and underground water. Overgrazing caused by the shrinking of grazing land causes compaction, which may lead to reduced infiltration rates. It is relevant to mention Lake Haramaya of eastern Ethiopia which disappeared, due to deforestation and clearing of land for farming activities in its surrounding watershed [44]. According to the Information obtained from interviews with key informants and focus groups, the volume of locally available streams and rivers and their flow patterns have decreased over time. The local farmers also mentioned that rainfall patterns have shown variability in both time and amount, and this has significantly affected the seasonal pattern of streams. As a result of this, women are forced to travel long distances, on average for two hours a day, to fetch water in dry seasons. All these indicate LULC change has direct implications on water resource availability and the magnitude of runoff and base flow in the watershed. This reasonably calls for the need for more effort on the balancing of the land use/cover change in general and investments in sustainable land management activities in particular so as to adjust the hydrologic-related disorder occurring in the watershed.

#### 3.3.3. Implications for Biodiversity

Land use/cover and habitat loss are widely known as the principal causes of biodiversity depletion in Ethiopia [45]. The changes in land use/cover also result in fragmentation of the landscape which in turn led to the loss of biodiversity as well as change in the structure and function of ecosystem services provision and human dependencies. According to the key informants, due to the absence of a clear forest tenure system, the forest trees of the catchment were indiscriminately destroyed. As a result, indigenous trees such as *Juniperous procera*, *Acacia caffra*, *Millettia ferugunea,* and *Ximenia ameriicana* which were once occupying the area are on the way to disappearing. Today, these indigenous trees are found only in protected areas, such as Church yard monasteries and inaccessible steeper mountainous areas. In many parts of the watershed, the indigenous trees are replaced by exotic trees such as eucalyptus. As was explained by the key informants, the decline of forest cover caused a decline in the number of wild animals. In some cases, animals such as tigers, lions, and antelopes which were commonly found in the watershed 30 years ago disappeared. Thus, the conversion of forest land to other type of land use caused numerous negative impacts on the ecosystem, as well as the livelihood of the society in the study area.

## 4. Conclusions

This study examined LULC changes, their driving forces, and implications on land resources between 1984 to 2017 in the Dedo watershed, southwest Ethiopia. Remote sensing and socioeconomic data sources were used as major input for the study. The results showed that there have been substantial changes in LULC in the Dedo watershed and its adjacent agroecosystem. The major changes were expansions of cultivation and settlement land and a decline in grazing, dense forest, and light vegetation land use/cover categories. The results indicated that dense vegetation land cover was among the most affected land cover with nearly 14.7 and 23.64% of its total area converted into other LULC types between the 1984 to 2000 and 2000 to 2017 periods, respectively. Cultivation land showed significant change over the entire analysis period with a nearly 15.7% increment. The study also indicated that socio-economic factors mainly population pressure and the establishment of resettlement programs were major driving forces in land use/cover change. Such a situation has critical implications for the deterioration of natural resources such as biodiversity loss, soil erosion, and a decline in the amount of water resources.

Conservation and management of natural resources in the study area are not adequate to alleviate the problem of local land degradation. As a result, the livelihood of the local community and the normal function of the ecosystem is under threat. This apparently tells us that it will continue to be a development challenge for the watershed and the nation at large. Thus, sustainable land management is vital to remove unsustainable practices and create a sustainable environment for all concerned. Therefore, overall watershed management is essential to safeguard an environment that will result in sustainable natural resource management and development in all dimensions of the study watershed.

## Figures and Tables

**Table 1:** Description of land use and land cover classes found in the study area.

Land use/land cover class	Description
Dense forest	Forest areas were covered with a dense growth of trees that formed nearly closed canopies.
Light vegetation	Areas covered with shrubs, bushes, and small trees, with useful wood, mixed with some grasses. In this study, this land cover class includes agricultural plantation (such as coffee and chat *enset*) horticultural plantation (such as fruits, ornamental shrubs, and trees, and vegetable gardens), and agrohorticultural plantation.
Grazing land	Grassy areas are used for grazing.
Cultivation land	Areas of land that is ploughed and/or prepared for raising both annual and perennial crops.
Settlement	Small rural communities and other built-up area.

**Table 2:** Land use land covers change (1984–2017) and their rate of change.

LULC class	*Land use land cover area coverage*						*Land use land cover change*		
	*1984*		*2000*		*2017*		*1984–2000*	*2000–2017*	*1984–2017*
	Hectare	%	Hectare	%	Hectare	%	Hectare	Hectare	Hectare
Cultivation land	21913.56	21.4	28925.48	28.22	37991.28	37.1	+7011.92	+9065.8	+16077.72
Grazing land	18050.58	17.6	17097.78	16.68	16151	15.72	−952.8	−946.78	−1899.58
Settlement	380.21	0.37	560.86	0.55	729.18	0.71	+180.65	+168.58	+348.97
Dense forest	23046.23	22.48	18395.3	17.95	13605	13.27	−4650.93	−4790.3	−9441.23
Light vegetation	39109.42	38.15	37520.58	36.6	34023.54	33.2	−1588.84	−3497.04	−5085.88
Total	102500	100	102500	100	102500	100			

**Table 3:** LULC transformation matrixes in different periods (area in hectare and %).

Changed from	Changed to	1984–2000 hectare	%	2000–2017 hectare	%
Cultivation land	Cultivation land	20648.4	94.22	26937.9	93.12
	Grazing land	518.77	2.36	907.26	3.14
	Settlement land	173.67	0.79	147.52	0.51
	Dense forest land	229.13	1.05	343.21	1.19
	Light vegetation land	343.53	1.57	589.59	2.04
	Total	21913.5	100	28925.48	100

Grazing land	Cultivation land	2090.06	11.60	2752.37	17.01
	Grazing land	15127.5	83.80	13426.71	78.53
	Settlement land	4.93	0.02	24.16	0.42
	Dense forest land	315.52	1.75	219.17	1.28
	Light vegetation land	512.57	2.84	675.3	3.95
	Total	18050.58	100	17097.78	100

Settlement land	Cultivation land	1.87	0.49	3.36	0.60
	Grazing land	0.00	0.00	0,00	0.00
	Settlement land	378.34	99.51	557.5	99.40
	Dense forest land	0.00	0.00	0.00	0.00
	Light vegetation land	0.00	0.00	0.00	0.00
	Total	380.21	100	560.86	100

Dense forest land	Cultivation land	3265.28	14.17	4348.91	23.64
	Grazing land	802.12	3.48	612	3.33
	Settlement land	0.00	0.00	0.00	0.00
	Dense forest land	17327.07	75.18	12296.25	66.84
	Light vegetation land	1651.76	7.17	1138.14	6.19
	Total	23046.23	100	18395.3	100

Light vegetation land	Cultivation land	2919.87	7.47	3948.72	10.52
	Grazing land	649.39	1.66	1205.03	3.21
	Settlement land	0.00	0.00	0.00	0.00
	Dense forest land	525.64	1.34	746.37	1.99
	Light vegetation land	35012.98	89.52	31620.46	84.27
	Total	39109.42	100	37520.58	100

## Data Availability

Readers can access the data supporting the conclusions of this study upon request to the corresponding author.
